# Functional metagenomics reveals novel antibiotic resistomes in polar soils

**DOI:** 10.1002/imt2.70069

**Published:** 2025-08-03

**Authors:** Xiuqin Xie, Weibin Cheng, Zhaohong Li, Rong He, Ke Yuan, Qinghua Zhang, Ruiqiang Yang, LiLi Ming, Ke Yu, Tiangang Luan, Baowei Chen

**Affiliations:** ^1^ Guangdong Provincial Key Laboratory of Marine Resources and Coastal Engineering School of Marine Sciences, Sun Yat‐sen University Zhuhai China; ^2^ Institute for Healthcare Artificial Intelligence Application The Affiliated Guangdong Second Provincial General Hospital of Jinan University Guangzhou China; ^3^ Faculty of Health Sciences City University of Macau Macao SAR China; ^4^ Guangdong Provincial Laboratory of Chemistry and Fine Chemical Engineering Jieyang Center Jieyang China; ^5^ State Key Laboratory of Environmental Chemistry and Ecotoxicology, Research Center for Eco‐Environmental Sciences Chinese Academy of Sciences Beijing China; ^6^ Technical Center of Gongbei Customs District Zhuhai China; ^7^ Eco‐environment and Resource Efficiency Research Laboratory, School of Environment and Energy, Shenzhen Graduate School Peking University Shenzhen China; ^8^ State Key Laboratory of Biocontrol, School of Life Sciences Sun Yat‐sen University Guangzhou China

## Abstract

Using a robust functional metagenomics approach, we demonstrated that polar environments are important reservoirs of novel antibiotic resistance genes (ARGs). DNA was initially extracted from cultured bacterial consortia in the polar soils and recombined into plasmid vectors and then transformed into *Escherichia coli* (*E. coli*) for the subsequent screening of antibiotic resistance. Consequently, we identified 671 novel polar ARGs with experimentally verified resistance against multiple clinical antibiotics (cefotaxime, folate synthesis inhibitors, and clindamycin). Bioinformatics analysis revealed that novel polar ARGs had limited mobility and dissemination potential and were seldom carried by human bacterial pathogens. Overall, this study offers a comprehensive perspective on previously overlooked novel ARGs in polar regions, advancing our understanding of environmental resistomes.

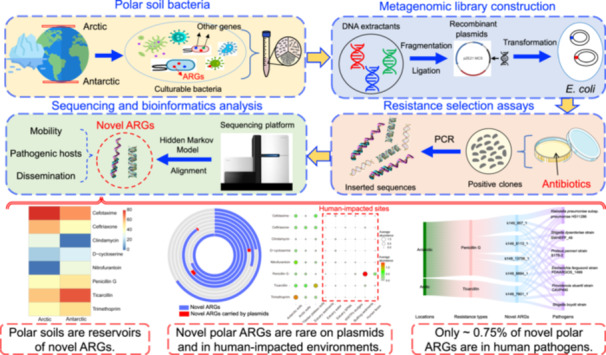

To the Editor,

Antibiotic resistance is a global health issue, with resistant bacterial pathogens causing hundreds of thousands of deaths annually [1, 2]. Many actual or potential human pathogens (e.g., *Serratia liquefariens* and *Yersinia enterorolztira*), which carry antibiotic resistance genes (ARGs), have been found in permanently frozen environments [3]. It has been estimated that approximately 4.0 × 10^21^ microbial cells are unprecedentedly released annually from frozen confinements due to global warming [4]. These glacial microbes may invade aquatic and terrestrial ecosystems [5, 6]. Undoubtedly, ARGs carried by ice‐entrapped microbes may also spread from the polar regions [7]. It has also been demonstrated that polar ARGs can be horizontally transferred and may eventually become a clinical concern [8].

Polar environments are important reservoirs of ARGs [9]. Our previous publications reported that cultured bacterial consortia from the polar soils were resistant to multiple clinical antibiotics, yet known ARGs associated with these antibiotics were absent from their genomes [9, 10]. This suggests that polar antibiotic resistomes remain incompletely characterized. Functional metagenomics employs heterologous expression of metagenomic DNA in surrogate hosts coupled with function‐based screening, high‐throughput sequencing, and bioinformatic analysis to discover genes with unclear functions from sequence data [11]. This approach enables in‐depth resistome exploration and can identify functionally verified ARGs independent of sequence similarity to known genes [12]. Owing to its advantages over traditional molecular biology assays and sequence‐based metagenomics (e.g., high throughput and functional verification independent of known gene annotation), functional metagenomics has successfully identified novel ARGs in diverse environmental samples [13]. Comprehensive characterization of novel ARGs in polar environments is critical to understanding their dissemination and mitigating health risks, e.g., incorporating new ARGs into genetics‐based diagnostics and targeted surveillance [14]. Here, we comprehensively explored novel ARGs in Arctic and Antarctic soils, assessing their mobility and dissemination, as well as potential host pathogenicity.

### Identification of diverse novel ARGs with experimentally verified functions in polar soils

Functional metagenomic selections permit the deep profiling of resistomes and can identify DNA fragments conferring resistance independent of known ARGs. We constructed eight metagenomic libraries (Antarctic, 0.05−2.1 Gb; Arctic, 0.07−0.29 Gb) by shotgun cloning 1.5 kb DNA fragments from cultured polar soil bacteria into *Escherichia coli* (Table S1), then screened them for resistance to 23 antibiotics across 9 drug categories (see methods in Supporting Information). Resistance was observed against 8 of the 23 tested antibiotics (Table S2), and resistance‐conferring DNA fragments were sequenced, assembled, and annotated (see methods in Supporting Information).

Among the 345,833 assembled open reading frames (ORFs), we annotated 18,657 as ARGs. Of these, 329 (Arctic) and 342 (Antarctic) ORFs met the reported criteria for novel ARGs (Figure 1A) [15, 16], representing low proportions of total polar ARGs (Figure S1A). Notably, ~20.0% of novel polar ARGs showed <80.0% identity to their closest National Center for Biotechnology Information (NCBI) homologs (Figure 1B). Approximately 70.0% of the novel ARGs conferred resistance to beta‐lactams (e.g., cefotaxime and ticarcillin), followed by folate synthesis inhibitors (~14.2%), d‐cycloserine (~6.4%), nitrofuran (~4.9%), and clindamycin (~4.8%). Novel ARG composition differed significantly between Arctic and Antarctic soils (two‐sided Fisher's exact test, *p* < 0.05) (Figure S1B).

**FIGURE 1:**
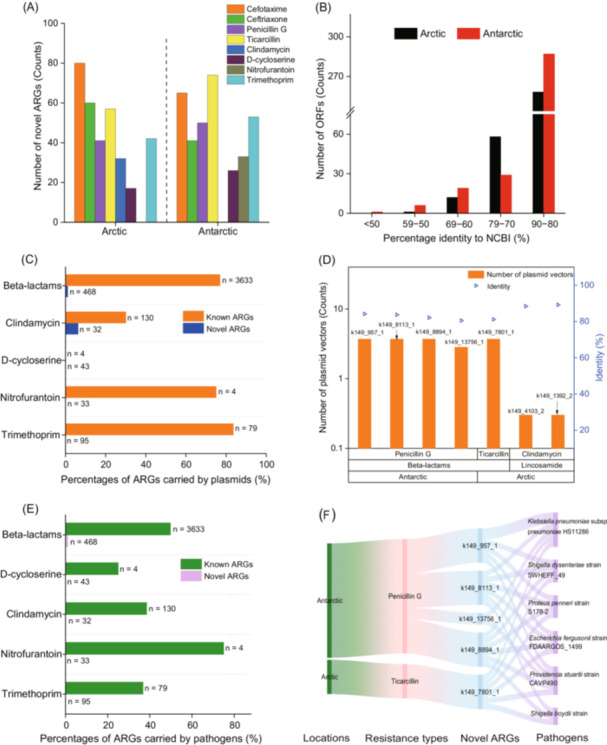
Novel antibiotic resistance genes (ARGs) conferring resistance to clinical antibiotics with unique characteristics in polar soils. (A) The number of novel ARGs categorized by antibiotic types in Arctic and Antarctic soils. (B) Amino acid identity between ORFs identified as novel polar ARGs (black, Arctic (329); red, Antarctic (342)) and their top hit in the NCBI protein database. (C) Comparisons in relative percentages of plasmid‐carrying ARGs over the total ARGs between novel and known ARGs. “*n*” represents the total number of ARG sequences used for alignment analysis. (D) Summary of novel polar ARGs carried by plasmids, including the number of plasmid vectors carrying novel ARGs, antibiotic categories, and the identities to known ARGs. A logarithm transformation of number of plasmid vectors was conducted. (E) Comparisons in relative percentages of human pathogenic bacteria‐carrying ARGs over the total ARGs between novel and known ARGs. “*n*” represents the total number of ARG sequences used for alignment analysis. (F) Predicted human pathogenic hosts of novel ARGs identified in Arctic and Antarctic soils.

### Low transferability of novel polar ARGs among bacterial hosts

Novel polar ARGs and known ARGs (from The Comprehensive Antibiotic Resistance Database) were both searched against the NCBI plasmid database and their potentials in horizontal gene transfer (HGT) among bacterial hosts were compared. At least 75.0% of known ARGs related to beta‐lactams, nitrofurantoin, and trimethoprim were plasmid‐associated, but novel polar ARGs were rare on plasmids (Figure 1C). Only seven novel polar ARGs were carried by plasmids, which were related to penicillin G (4), ticarcillin (1), and clindamycin (2). Notably, the penicillin G (beta‐lactam) resistance genes occurred on >690 plasmid types (Figure 1D). For beta‐lactam resistance genes (BRGs), the occurrence of known ARGs encoding Class A beta‐lactamases on plasmids was statistically significantly higher than that of novel polar ARGs (*p* < 0.05) (Figure S2).

### Low occurrence of novel polar ARGs in pathogenic bacterial genomes

We analyzed both novel polar ARGs and aforementioned known ARGs against a pathogenic bacterial database and compared their occurrence patterns. Known ARGs carried by pathogenic hosts represented all categories of the identified novel polar ARGs, comprising >25.0% of known ARGs in each category (Figure 1E). In contrast, only 0.75% of the novel polar ARGs could be found in the genomes of pathogenic bacteria, all conferring resistance to beta‐lactams. Notably, each of the four novel ARGs was found in six pathogenic strains, including *Klebsiella pneumoniae*, *Shigella dysenteriae*, *Shigella boydii*, *Proteus penneri*, *Escherichia fergusonii*, and *Providencia stuartii* (Figure 1F). These pathogen‐associated novel ARGs were plasmid‐borne (Figure 1D,F). With respect to BRGs, occurrence frequency and the composition of pathogenic hosts showed statistically significant differences between novel and known ARGs according to the Kruskal–Wallis test (*p* < 0.05) (Figure S3A,B).

### Limited dissemination of novel polar ARGs to human‐impacted environments

We analyzed novel polar ARGs in metagenomes from diverse environments (Table S3), including representative pollution sources of ARGs due to antibiotic use (e.g., wastewater treatment plants and aquaculture farms) and relatively pristine regions with minimal anthropogenic impacts (e.g., the Tibetan Plateau). We detected 138 novel polar ARGs across 9 metagenomic datasets, with total abundance ranging from ND (no detected) (D1, estuary sediment) to 11.8 ARG‐like reads in *per* million sequencing reads (S2, Antarctic soil) (Figure 2A). Beta‐lactams‐related (65.2%) and trimethoprim‐related (15.2%) ARGs were most abundant among them. Novel polar ARGs were more prevalent in pristine versus human‐impacted environments (Figure 2A). Tibetan Plateau soils contained 60 novel ARGs, compared to just 29 across seven human‐impacted sites. In the same way, we analyzed known ARGs in matching categories of novel polar ARGs. Novel ARGs outnumbered known ARGs in pristine environments (*p* < 0.05; Figure 2B), and the reverse pattern was observed in human‐impacted settings (Figure 2C). Principal component analysis revealed a statistically significant separation between pristine and human‐impacted environments based on novel ARG profiles (ANOSIM, *p* < 0.05; Figure 2D).

**FIGURE 2:**
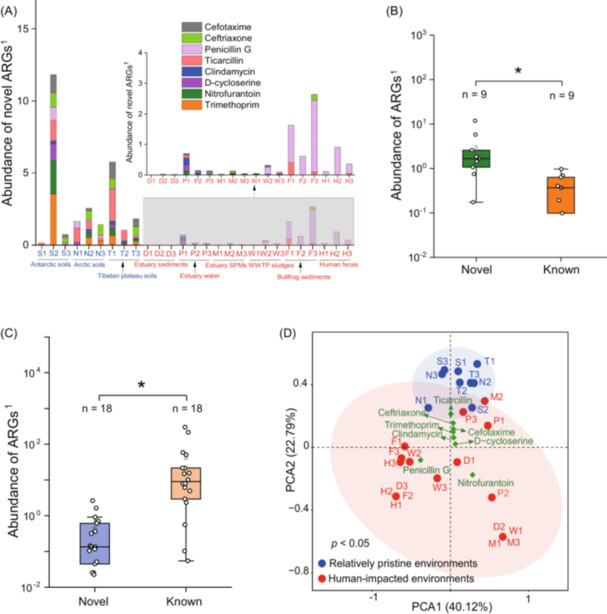
Novel antibiotic resistance genes (ARGs) identified in polar soils are popular in natural environments instead of human‐impacted ones. (A) Abundance of novel polar ARGs in different environmental settings with varying human impacts, and S1, S2, etc. represent sampling sites from different environments. Comparison in the total abundance between novel and known ARGs in the relatively pristine (B) and human‐impacted (C) environments, where “*” represents a statistically significant difference by Kruskal–Wallis test (*p* < 0.05) and “*n*” means the number of sequencing datasets used for this analysis. “1” indicates the unit of ARG abundance as one ARG‐like read *per* million sequencing reads. (D) Categorization of sampling sites according to the abundance of novel polar ARGs using the principal components analysis (PCA) method.

Natural environmental niches free of anthropogenic impacts are widely recognized as major ARG reservoirs [17]. Nevertheless, the vast functional diversity of environmental resistomes is always underestimated, as the identified ARGs in soils are largely dissimilar from ARGs in public repositories [16]. Twenty‐four novel tetracycline resistance genes were identified in soil samples from pristine (antibiotic‐free) sites in Yunnan, Sichuan, and Tibet, China [13]. New sulfonamide resistance genes were discovered in forest soils with no history of exposure to synthetic drugs [18]. This study confirms the presence of novel, functionally verified ARGs associated with clinical antibiotics in polar soils. Moreover, the discovery of novel ARGs underscores the limitations of conventional metagenomics approaches that rely exclusively on public databases for comprehensive interrogation of environmental resistomes. Of the antibiotics used for resistance screening in this study, penicillin G and d‐cycloserine may be naturally produced by microorganisms, while the others are synthetic or semi‐synthetic. Novel ARGs conferring resistance to synthetic or semi‐synthetic antibiotics were also found in our polar samples. Our findings suggest that indigenous microbial genomes in natural environments serve as a genetic reservoir for resistance development and dissemination, even against synthetic antimicrobials.

Our results demonstrated that novel ARGs identified in polar soils had limited plasmid‐mediated transferability among bacterial hosts. This trait is also possessed by novel ARGs found in other environments. For example, novel ARGs identified in agricultural and grassland soils collected from the United States of America exhibited no association with mobile genetic elements (MGEs) [16], and pre‐antibiotic‐era microflora (e.g., Enterobacteriaceae) rarely carried ARGs on MGEs [19]. Furthermore, known ARGs detected in pristine environments exhibit similarly low transferability, as demonstrated by the scarcity of plasmid‐associated ARGs in Tibetan ecosystems [20]. In contrast, plasmid‐borne ARGs are far more abundant in human‐impacted environments, consistent with previous studies [9, 10]. These findings imply that the absence of selective pressures may contribute to a key characteristic of pristine‐environment resistomes: exceptionally low ARG mobility.

Environmental ARG reservoirs are implicated as the sources of antibiotic resistance for human pathogens [15]. In this study, only a small fraction of novel polar ARGs (~0.75%) were potentially detectable in human pathogens, suggesting minimal current health risks posed by these novel ARGs. It was previously reported that only 1 of 2895 soil ARGs matched pathogen‐associated genes at 100% identity [16]. Notably, the detection of novel ARGs in the genomes of human pathogens suggests that exchange barriers between environmental reservoirs and human pathogens may be overcome. ARGs in human pathogens exhibit greater HGT potential than those in non‐pathogens [16], as observed in this study, which can in turn facilitate their transmission between human pathogens. The acquisition of novel ARGs by bacterial pathogens can enhance antibiotic resistance, potentially raising unforeseen clinical burdens.

Novel ARGs identified in polar soils exhibited low occurrence in human‐impacted environments, reflecting their limited dissemination. In contrast, known ARGs from the same categories as novel polar ARGs were consistently enriched in the environments subjected to significant anthropogenic impacts, consistent with previous studies [9, 10, 20]. Interestingly, novel polar ARGs were also prevalent and abundant in the Tibetan plateau soils despite their geographical distance from polar regions. Collectively, these results demonstrate that novel polar ARGs significantly contribute to distinguishing native antibiotic resistomes from human‐impacted ones. This study suggests that novel polar ARGs may serve as useful markers for characterizing ARG profiles in pristine circumstances devoid of human impacts.

This study demonstrates that polar environments are important reservoirs of novel ARGs. The novel ARGs identified in polar soils exhibit limited mobility, dissemination potential, and pathogenic risks. This study also highlights that metagenomics investigations of known ARGs may overlook enormous yet‐to‐be‐identified ARGs, thereby underestimating the diversity of antibiotic resistomes in natural environments.

Detailed experimental materials and procedures, including sample collection and processing techniques, and statistical analysis approaches are described in the Supporting Information.

## AUTHOR CONTRIBUTIONS

**Xiuqin Xie**: Writing—original draft; writing—review and editing; methodology; data curation; formal analysis; validation; visualization; funding acquisition. **Weibin Cheng**: Conceptualization; formal analysis; writing—review and editing. **Zhaohong Li**: Methodology; data curation; formal analysis; writing—review and editing. **Rong He**: Methodology; data curation; formal analysis; validation. **Ke Yuan**: Methodology; writing—review and editing. **Qinghua Zhang**: Investigation; resources; writing—review and editing. **Ruiqiang Yang**: Investigation; writing—review and editing; resources; funding acquisition. **LiLi Ming**: Data curation; formal analysis; methodology; writing—review and editing; funding acquisition. **Ke Yu**: Methodology; data curation; formal analysis; validation; writing—review and editing. **Tiangang Luan**: Conceptualization; writing—review and editing. **Baowei Chen**: Conceptualization; writing—review and editing; methodology; data curation; formal analysis; supervision; validation; writing—original draft; visualization; funding acquisition; project administration.

## CONFLICT OF INTEREST STATEMENT

The authors declare no conflicts of interest.

## ETHICS STATEMENT

No animals or humans were involved in this study.

## Supporting information


**Figure S1.** Relative percentages of novel ARGs over the total annotated ARGs and for different antibiotic classes.
**Figure S2.** Number of plasmid vectors for novel and known ARGs related to Class A beta‐lactams.
**Figure S3.** Significant differences exist in both the number and compositional patterns of human bacterial pathogen hosts between novel and known Class A beta‐lactamase‐encoding ARGs.

**Table S1.** Summary of information regarding functional metagenomic libraries constructed from the polar soil samples.
**Table S2.** Resistance tests of the constructed clone libraries to 23 antibiotics in Arctic and Antarctic soils.
**Table S3.** Information regarding sequencing datasets used in this study.

## Data Availability

The data that support the findings of this study are available on request from the corresponding author. The data are not publicly available due to privacy or ethical restrictions. All the sequencing data have been deposited in the NCBI Sequence Read Archive (SRA) database (https://www.ncbi.nlm.nih.gov/sra/?term=PRJNA1206728). The data and scripts used are saved in GitHub (https://github.com/99amber/IMETA-2025-579-all-data-and-scripts). All other data are available in the main text or supporting information. Supplementary materials (methods, figures, tables, graphical abstract, slides, videos, Chinese translated version, and update materials) may be found in the online DOI or iMeta Science (http://www.imeta.science/).
