# Supplementary material for: Functional metagenomics reveals novel antibiotic resistomes in polar soils
Source: Imeta. 2025 Aug 3;4(4):e70069. doi: 10.1002/imt2.70069 (PMC12371247; doi:10.1002/imt2.70069)
Supplement: Supplementary file 1 — Figure S1. Relative percentages of novel ARGs over the total annotated ARGs and for different antibiotic classes. Figure S2. Number of plasmid vectors for novel and known ARGs related to Class A beta‐lactams. Figure S3. Significant differences exist in both the number and compositional patterns of human bacterial pathogen hosts between novel and known Class A beta‐lactamase‐encoding ARGs. [file IMT2-4-e70069-s002.docx]

**Supporting information to**

**Functional Metagenomics Reveals Novel Antibiotic Resistomes in Polar Soils**

**Running title:** New Antibiotic Resistomes Identified in Polar Soils

Xiuqin Xie^1#^, Weibin Cheng^2,3#^, Zhaohong Li^1^, Rong He^1^, Ke Yuan^1, 4^, Qinghua Zhang^5^, Ruiqiang Yang^5^, LiLi Ming^6^, Ke Yu^7^, Tiangang Luan^4,8^, Baowei Chen^1, 4*^

^1^Guangdong Provincial Key Laboratory of Marine Resources and Coastal Engineering, School of Marine Sciences, Sun Yat-sen University, Zhuhai, 519082, China

^2^Institute for Healthcare Artificial Intelligence Application, The Affiliated Guangdong Second Provincial General Hospital of Jinan University, Guangzhou, 510275, China

^3^Faculty of Health Sciences, City University of Macau, Macao SAR, 999078, China

^4^Guangdong Provincial Laboratory of Chemistry and Fine Chemical Engineering Jieyang Center, Jieyang 515200, China

^5^State Key Laboratory of Environmental Chemistry and Ecotoxicology, Research Center for Eco-Environmental Sciences, Chinese Academy of Sciences, Beijing, 100085, China

^6^Technical Center of Gongbei Customs District, Zhuhai, 519000, China

^7^Eco-environment and Resource Efficiency Research Laboratory, School of Environment and Energy, Shenzhen Graduate School, Peking University, Shenzhen, 518055, China

^8^State Key Laboratory of Biocontrol, School of Life Sciences, Sun Yat-sen University, Guangzhou, 510275, China

^#^These authors contributed equally: Xiuqin Xie, Weibin Cheng

^*^Correspondence: [chenbw5@mail.sysu.edu.cn](mailto:chenbw5@mail.sysu.edu.cn) (Baowei Chen)

**METHODS**

**Sample collection**

A total of 4 soil samples (S1, S2, S3, and S4) were collected from Antarctic in January 2015, and another four soil samples (N1, N2, N3, and N4) were collected from Arctic in August 2015. Site S1 and S2 are located at the Antarctic Penguin Island that is a seabird habitat designated as an Antarctic Special Area of Conservation. Site S3 is situated at the west seacoast. Among all sampling sites in the South Polar region, Site S4 is closest to the Chinese Great Wall Station, and lies directly to its due south. Another four soil samples (N1, N2, N3, and N4) were collected from the Kongsfjorden in the North Pole, which is one of the largest fjords on Spitsbergen. At each of sampling sites, the top 5-cm layer of soils was taken from 5 different positions using a sterile sampling shovel, and the samples were thoroughly mixed and immediately stored in the sterilized centrifuge tubes. The collected samples were kept at 4 °C for ~ 2 weeks until the following treatments.

In this study, some auxiliary metagenomes of other environmental settings were used to investigate the occurrence of novel ARGs identified in polar soils, including 3 soil samples (S1, S2, and S3) from Antarctic in 2015, 3 samples (N1, N2, and N3) collected from Arctic in 2015, 3 soil samples (T1, T2, and T3) from the Shergyla Mountain, Tibetan Plateau in 2019; activated sludges (W1, W2, and W3) collected from a wastewater treatment plant (WWTP) in Beijing, China in 2022; 3 sediment samples (D1, D2, and D3), 3 water samples (P1, P2, and P3), and 3 suspended particulate matters (SPMs) (M1, M2, and M3), which were all collected from the Pearl River Estuary (PRE) in 2019; 3 sediments (F1, F2, and F3) collected from a bullfrog farm in Shantou, China in 2015, and 3 human feces samples downloaded from NCBI SRA database (H1, H2, and H3). Detailed information regarding sampling sites and metagenomic datasets, including sample locations, coordinates, and accession numbers, is tabulated in Table S3.

**Functional verification of DNA fragments**

## Metagenomic library construction

Soil samples (~ 1.0 g) were cultivated with Luria-Bertani (LB) medium (9.0 mL), and incubated overnight on a horizontal rotator with a rate of 150 rpm at 4 °C. DNA were extracted from culturable bacterial consortiums using OMEGA bacteria DNA kit (Omega, Norcross, GA, USA) following the manufacturer’s protocol. The extracted DNA was measured using a Thermo Scientific NanoDrop2000 Spectrophotometer. Then the metagenomic DNA was fragmented into a size of about 1−5 kb using the M220 shearing instrument (Covaris, Woburn, MA, USA) with the manufacturer’s recommended settings using the following parameters: Peak Incident Power = 6, Duty Factor = 20%, Cycles per Burst = 1000, Treatment Time = 1800 s. Sheared DNA was size-selected by agarose gel electrophoresis (1.0%) and DNA corresponding to the length of 2−4 kb was excised and extracted using a QIAquick Gel Extraction kit (Qiagen, Hilden, Germany) following the manufacturer’s manual. The recovered DNA was then end-repaired using the End-It DNA End Repair kit (Epicentre, Madison, WI, USA) with the following steps:

1. Each aliquot of 50 μL reaction system included the following: 5 μL 10X End-Repair Buffer, 5 μL dNTP mix (2.5 mM), 5 μL ATP (10 mM), 1 μL End-Repair Enzyme Mix, and x μL nuclease-free H_2_O to a final volume of 50 μL. The standard 50 µL reaction will end-repair up to 5 μg of DNA.
2. Incubating at ambient temperature for 45 min.
3. Stopping the reactions by heating at 70 °C for 15 min.

End-repaired DNA was then purified with the QIAquick PCR Purification kit (Qiagen) according to the recommended protocol. The quality and quantities of recovered DNA were determined using a Thermo Scientific NanoDrop2000 Spectrophotometer.

The pZE21 vectors were linearized at the HincⅡ site using the PCR with the AccuPrime™ Pfx SuperMix polymerase (Thermo Fisher), forward primer (5'-GACGGTATCGATAAGCTTGAT-3') and reverse primer (5'-GACCTCGAGGGGGGG-3'). Linearized pZE21 was then size-selected (~2200 bp) by agarose gel electrophoresis (1.0%) and purified as described above. Pure vectors were dephosphorylated using Quick CIP kit (NEB #M0525) with following reaction system:

1. Each 20-μL aliquot of reaction system included: CutSmart Buffer (10 ×) = 2 μL, CIP = 1 μL, linearized pZE21 up to 1 μg, and x μL nuclease-free H_2_O to adjust a final volume to 20 μL.
2. Incubating at 37 °C for 10 min.
3. Terminating the reactions by heating-inactivation at 80 °C for 2 min.

The recovered linearized pZE21 DNA was then purified with the QIAquick PCR Purification kit (Qiagen) using the recommended manuals. The quality and quantities of recovered DNA were determined using a Thermo Scientific NanoDrop2000 Spectrophotometer. End-repaired DNA fragments and linearized vectors were then ligated together using Fast-Link™ DNA Ligation Kits (Epicentre, USA) at a mass ratio of 5:1 (inserted DNA: vectors) following the manufacturer’s protocol (the insert and vector were similarly sized and the mass ratio approximates a molar ratio). The specific process is as follows:

1. The reaction was assembled in a microcentrifuge tube at room temperature as listed: 1.5 μL 10 × Fast-Link buffer, 0.75 μL ATP (10 mM), 1 μL Fast-Link DNA Ligase, 5:1 mass ratio of metagenomic DNA to vector, and nuclease-free H_2_O to a final reaction volume of 15 μL.
2. The reaction was conducted at room temperature overnight.
3. The reaction was performed in a water bath or heat block at 70 °C for 15 min to inactivate the Fast-Link DNA Ligase.

Following heat inactivation, ligations were dialyzed for 30 min using a 0.025 μm cellulose membrane (Millipore catalogue number VSWP09025). Electroporation was conducted using the manufacturer’s recommendation protocols (Bio-Rad, Munich, Germany) and the following electroporation conditions: 20 kV, 200 Ω, and 25 µF. The transformed bacterial cells were recovered in 1.0 mL SOC broth at 37 °C with a rate of 150 rpm for 1 h. After incubation, the culture was diluted by10 folds, and a 100-μL aliquot was plated onto the LB agar containing 50 μg/mL kanamycin. For each of libraries, insert size distribution was estimated by gel electrophoresis of PCR products, which were obtained by amplifying the inserts from 10 randomly picked clones using the primers flanking the *Hinc*II site of the multiple cloning site of the pZE21 MCS 1 vector (which possesses a selectable marker for kanamycin resistance) [1]. Library size was estimated by multiplying the average PCR-based insert size by the number of titred colony forming units (CFUs) after transformation recovery. The rest of the recovered bacteria were inoculated into 20 mL LB broth containing 50 μg/mL kanamycin, and incubated for 12−16 h with a rate of 150 rpm at 37 °C. The final cultures were frozen down with 10.0% glycerol and kept at 80 °C for the following resistance screening. In this study, we constructed 8 metagenomic DNA libraries from Arctic soils (4) and Antarctic soils (4), with an average insert size of 1.5 kb. The total library size of Antarctic soils was about 3.0 Gb with a range from 0.05 to 2.1 Gb, and that of Arctic soils was approximately 0.6 Gb (range: 0.07−0.29 Gb) (Table S1).

## Antibiotic resistance screening

Each metagenomic library was plated on the LB agar containing both 50 μg/mL kanamycin *per* above and one of 23 individual antibiotics (at concentrations indicated in Table S2) for functional selection on antibiotic resistance, which represented 9 classes of antibiotics commonly used for clinical treatment. These antibiotics included beta-lactams (amoxicillin, aztreonam, cefazolin, ceftriaxone, cefepime, cefotaxime meropenem, imipenem, penicillin G, and ticarcillin), tetracyclines (tetracycline and tigecycline), aminoglycosides (gentamicin), chloramphenicol antibiotic (chloramphenicol), lincosamide antibiotic (clindamycin), amino acid derivatives (D-cycloserine), polypeptide antibiotics (colistin), nitrofuran (nitrofurantoin), and folate synthesis inhibitor antibiotics (trimethoprim, cotrimoxazole, rifampicin, ciprofloxacin, and moxifloxacin) (Table S2). After plating, antibiotic selections were incubated at 37 °C for 18 h to allow the growth of clones carrying an antibiotic-resistant DNA insert sequence. After overnight growth, all colonies were collected by adding 750 μL of 20% LB-glycerol to the plates and scraping with L-shaped cell scraper to gently remove from the plates. All bacterial cells were then harvested into 2 mL LB-kanamycin with 20% glycerol and stored at -80 °C prior to PCR amplification of metagenomic fragments enabling the resistance to concerned antibiotics. Our results showed that metagenomic libraries established from Arctic soils were associated with the resistance to 8 antibiotics (*i.e.*, cefotaxime, ceftriaxone, penicillin G and ticarcillin (beta-lactams), clindamycin (lincosamide), D-cycloserine (amino acid derivatives), nitrofurantoin (nitrofuran), and trimethoprim (folate synthesis inhibitors)), and those of Antarctic soils were resistant to 7 antibiotics including the aforementioned antibiotics except for clindamycin.

## Amplification of inserted metagenomic DNA fragments related to antibiotic resistance

Freezer stocks of antibiotic resistant transformants were thawed, and 300 μL of cells were pelleted by centrifugation at 13,000 rpm for 2 min. The pelleted cells were gently washed with 1.0 mL of nuclease-free H_2_O. Cells were subsequently pelleted again and re-suspended in 30 μL nuclease-free H_2_O. Re-suspensions were then frozen at -20 °C for 1 h and thawed to result in cell lysis. The thawed re-suspension was pelleted by centrifugation at 13,000 rpm for 2 min, and the resulting supernatants were used as the templates for PCR amplification of resistance-conferring DNA fragments. The PCR reaction solution consisted of 2.5 μL of templates, 2.5 μL of ThermoPol reaction buffer (New England Biolabs), 0.5 μL of 10 μM deoxynucleotide triphosphates (dNTPs, New England Biolabs), 0.5 μL of Taq polymerase (5 U/mL), 3 μL of a customed primer mix, and 16 μL of nuclease-free H_2_O to bring the final volume to 25 μL. The customed primer mix contained 3 forward and 3 reverse primers, each targeting the DNA sequence flanking the *Hinc*II site in the pZE21 MCS 1 vector and staggered by one base pair. The staggered primer mix could guarantee diverse nucleotide composition during early Illumina sequencing cycles, which was comprised of the following primer volumes (from a 10 μM stock) in a single PCR reaction: (primer F1, 5'-CCGAATTCATTAAAGAGGAGAAAG, 0.5 μL); (primer F2, 5'-CGAATT CATTAAAGAGGAGAAAGG, 0.5 μL); (primer F3, 5'-GAATTCATTAAAGAG GAGAAAGGTAC, 0.5 μL); (primer R1, 5'-GATATCAAGCTTATCGATACCGTC, 0.21 μL); (primer R2, 5'-CGATATCAAGCTTATCGATACCG, 0.43 μL); (primer R3, 5'-TCGATATCAAGCTTATCGATACC, 0.86 μL). PCR amplification reactions were then carried out under the following thermocycler conditions: 95 °C for 10 min, 25 cycles of 94 °C for 5 min + 55 °C for 45 s + 72 °C for 5.5 min and 72 °C for 10 min. Thereafter, the amplified metagenomic inserts were cleaned using the Qiagen QIAquick PCR purification kit and quantified using the Thermo Scientific NanoDrop2000 Spectrophotometer.

**Sequencing and bioinformatic analysis**

The amplified metagenomic inserts from each antibiotic selection were delivered to the headquarter of Annoroad Gene Technology Co., Ltd, Beijing, China, and sequenced using Illumina HiSeq 2500 platform. In brief, DNA was sheard into fragments approximately 350 nucleotides in length using the Annoroad^®^ Universal DNA Fragmentase kit V2.0. The overhangs of these DNA fragments were end-repaired using T4 DNA polymerase, Klenow Fragment and T4 Polynucleotide Kinase in turn. A single ‘A’ base was connected to the 3′ end of the blunt DNA fragments, followed by ligation of adapters to the DNA fragment ends. Index tags were incorporated into the adapters for constructing DNA libraries. The DNA fragments were purified via gel-electrophoresis, and subsequently enriched and amplified through PCR, during which the index tags were introduced into the adapter. The qualified DNA libraries were used for sequencing using the Illumina HiSeq 2500 platform. Approximately, 2.0 Gb of pair-end reads (PE150) was achieved for each of DNA samples.

Raw sequencing reads were screened for obtaining the high-quality reads with q value being higher than 30 and *p* value being greater than 50. The clean reads were filtered to remove the host *E. coli* strain DH10B and vector sequences using the Bowtie (v 2-2.2.1) [2], SAMtools (v 1.8) [3], bedtools (v 2-2.25.0) [4] and Crossmatch [5]. The acquired clean reads were assembled using MEGAHIT (v 1.1.1) [6] with the following parameters: --k-step 10 --k-min 33 --k-max 149 -min-contig-len 500. Following sequencing and assembling of the resulted antibiotic resistance clones, a total of 61374 and 99274 contigs (> 500 bp) were recovered from Arctic and Antarctic soils, respectively. The contigs obtained from screening of beta-lactams could account for ~ 70.0% of the total contigs recovered from Arctic soils, followed by clindamycin (11.0%), trimethoprim (9.4%), D-cycloserine (9.1%), and nitrofurantoin (0.4%). With respect to Antarctic soils, relative percentages of antibiotic resistance contigs were 59.8% (beta-lactams), 15.0% (nitrofurantoin), 12.6% (D-cycloserine), and 12.5% (trimethoprim). A significant difference in the compositional pattern of retrieved ARG contigs sorted by antibiotic screening was observed between Arctic and Antarctic soils (two-sided Fisher’s exact test, *p* < 0.01). After assemblying, open reading frames (ORF) were predicted using Prodigal v 2.6.2 [7], and the translated ORFs were annotated by searching the amino acid sequences against the resistance-gene-specific profile HMMs databases ([*http://dantaslab.wustl.edu/resfams*](http://dantaslab.wustl.edu/resfams)) with HMMER3 [8]. The HMMER3 was run with the option ‘‘--cut_ga’’, requiring that genes meet profile specific gathering thresholds (rather than a global, more permissive, default log odds cutoff) before annotation [1]. An ORF from a resistance-conferring DNA fragment was identified as an ARGs if it exceeded the stringent, profile-specific gathering thresholds defined by the custom-built set of profile HMMs. Percentage identity comparisons of all screened ARGs were conducted using a BlastX query against the NCBI protein Non-Redundant (NR) database (retrieved August 2022). An ORF was classified as “known ARGs” if it had more than 90% identity to the closest homologs in the NR database. On the contrary, an ORF was identified as “novel ARGs” if it had the identity less than or equal to 90% and a coverage greater than 65% [9,10]. Examples of novel ARGs include those exhibiting high sequence divergence from known genes, those whose resistance function cannot be predicted from sequence data, bifunctional resistance genes, and those employing unconventional resistance mechanisms [11]. Besides, the number of ORFs annotated as ARGs in the Arctic and Antarctic metagenomic libraries was 5853 and 12804, respectively. A total of 329 (Arctic) and 342 (Antarctic) ORFs were identified as novel ARGs and most of these novel ARGs had the genetic similarity of 80.0%−90.0% to the best-hit reference genes in the NCBI non-redundant (NR) protein database, and about 5.7% of them had a low identity (< 70.0%).

To investigate the mobility and pathogenicity of novel ARGs in polar soils, the screened novel ARGs were aligned against the plasmid and pathogens database. A total of 85,546 plasmid sequences were downloaded from the NCBI database in 2024. A list of NCBI taxonomy IDs for human pathogenic bacteria was obtained from the publication with an ‘Environment’ label of ‘‘Human’’ and a ‘Pathogenicity’ label of ‘‘Pathogen’’ [1]. Human pathogenic bacteria database was downloaded and constructed according to this list. The novel ARGs were determined to be possibly carried by plasmids or human pathogenic bacteria and to occur in other environments if the identities was greater than 90% and the sequence coverages were greater than 90%.

**Statistical analysis**

Independent samples *t*-tests and Kruskal-Wallis tests were performed using IBM SPSS Statistics 23 for Windows. Fisher’s exact test was conducted in RStudio (Version 2025.05.1+513) [12] with the parameter simulate.p.value = TRUE. ANOSIM analysis, which could evaluate the differences among Bray-Curtis distance matrices with the permutations number being 999 was performed in RStudio. Statistical significance was defined as *p* < 0.05.

**REFERENCES**

1. Forsberg, Kevin J., Sanket Patel, Molly K. Gibson, Christian L. Lauber, Rob Knight, Noah Fierer, Gautam Dantas. 2014. “Bacterial phylogeny structures soil resistomes across habitats.” *Nature* 509: 612. <https://doi.org/10.1038/nature13377>

2. Langmead, Ben, Steven L Salzberg. 2012. “Fast gapped-read alignment with Bowtie 2.” *Nature Methods* 9: 357-359. <https://doi.org/10.1038/nmeth.1923>

3. Li, Heng, Bob Handsaker, Alec Wysoker, Tim Fennell, Jue Ruan, Nils Homer, Gabor Marth, Goncalo Abecasis, Richard Durbin. 2009. “The sequence alignment/map format and SAMtools.” *Bioinformatics* 25: 2078-2079. <https://doi.org/10.1093/bioinformatics/btp352>

4. Quinlan, Aaron R, Ira M Hall. 2010. “BEDTools: a flexible suite of utilities for comparing genomic features.” *Bioinformatics* 26: 841-842. <https://doi.org/10.1093/bioinformatics/btq033>

5. Ewing, Brent, Phil Green. 1998. “Base-calling of automated sequencer traces using phred. II. Error probabilities.” *Genome Research* 8: 186-194. <https://doi.org/10.1101/gr.8.3.186>

6. Li, Dinghua, Chi-Man Liu, Ruibang Luo, Kunihiko Sadakane, Tak-Wah Lam. 2015. “MEGAHIT: an ultra-fast single-node solution for large and complex metagenomics assembly via succinct de Bruijn graph.” *Bioinformatics* 31: 1674-1676. <https://doi.org/10.1093/bioinformatics/btv033>

7. Hyatt, Doug, Gwo-Liang Chen, Philip F Locascio, Miriam L Land, Frank W Larimer, Loren J Hauser. 2010. “Prodigal: prokaryotic gene recognition and translation initiation site identification.” *Bioinformatics* 11: 119. <https://doi.org/10.1186/1471-2105-11-119>

8. Gibson, Molly K., Kevin J. Forsberg, Gautam Dantas. 2015. “Improved annotation of antibiotic resistance determinants reveals microbial resistomes cluster by ecology.” *The ISME Journal* 9: 207-216. <https://doi.org/10.1038/ismej.2014.106>

9. Forsberg, Kevin J., Alejandro Reyes, Bin Wang, Elizabeth M. Selleck, Morten O. A. Sommer, Gautam Dantas. 2012. “The shared antibiotic resistome of soil bacteria and human pathogens.” *Science* 337: 1107-1111. <https://doi.org/10.1126/science.1220761>

10. Razavi, Mohammad, Nachiket P. Marathe, Michael R. Gillings, Carl-Fredrik Flach, Erik Kristiansson, D. G. Joakim Larsson. 2017. “Discovery of the fourth mobile sulfonamide resistance gene.” *Microbiome* 5: 160. <https://doi.org/10.1186/s40168-017-0379-y>

11. Pehrsson, Erica C., Kevin J. Forsberg, Molly K. Gibson, Sara Ahmadi, Gautam Dantas. 2013. “Novel resistance functions uncovered using functional metagenomic investigations of resistance reservoirs.” *Frontiers in Microbiology* 4: 145. <https://doi.org/10.3389/fmicb.2013.00145>

12. Racine, Jeffrey S. 2012. “RStudio: a platform-independent IDE fro R and SWEAVE.” *Journal of Applied Econometrics* 27: 167-172. <https://doi.org/10.1002/jae.1278>

**Figure S1** Relative percentages of novel ARGs over the total annotated ARGs (A) and for different antibiotic classes (B), ‘*’ represents a significant difference by two-sided Fisher's exact test (*p* < 0.05).

**Figure S2** Number of plasmid vectors for novel and known ARGs related to Class A beta-lactams, where a logarithm transformation was conducted. ‘*’ represents a significant difference between novel and known ARGs by Kruskal-Wallis test (*p* < 0.05), and ‘n’ represents the total number of ARG sequences used for alignment analysis.

**Figure S3** Significant differences exist in both the number and compositional patterns of human bacterial pathogen hosts between novel and known Class A beta-lactamase-encoding ARGs. (A) Comparison in detection frequencies of human bacterial pathogens between novel and known ARGs encoding Class A beta-lactamases, where a logarithm transformation of detection frequencies was carried out; (B) Compositional patterns of pathogenic hosts carrying novel and known ARGs expressing Class A beta-lactams, respectively.
